# Time-Dependent Effect of Anthracycline-Based Chemotherapy on Central Arterial Stiffness: A Systematic Review and Meta-Analysis

**DOI:** 10.3389/fcvm.2022.873898

**Published:** 2022-07-05

**Authors:** Caroline Schneider, Nathalia González-Jaramillo, Thimo Marcin, Kristin L. Campbell, Thomas Suter, Arjola Bano, Matthias Wilhelm, Prisca Eser

**Affiliations:** ^1^Department of Cardiology, Inselspital, Bern University Hospital, University of Bern, Bern, Switzerland; ^2^Graduate School for Health Sciences, University of Bern, Bern, Switzerland; ^3^Institute of Social and Preventive Medicine (ISPM), University of Bern, Bern, Switzerland; ^4^Faculty of Medicine, University of British Columbia, Vancouver, BC, Canada

**Keywords:** vasculotoxicity, aortic distensibility, pulse-wave-velocity, breast cancer, lymphoma

## Abstract

**Background and Aims:**

Anthracycline-based chemotherapy (ANTH-BC) has been proposed to increase arterial stiffness, however, the time-dependency of these effects remain unclear. This systematic review and meta-analysis aimed to investigate the time-dependent effect of ANTH-BC on markers of central aortic stiffness, namely aortic distensibility (AD) and pulse-wave-velocity (PWV) in cancer patients.

**Methods:**

An extensive literature search without language restrictions was performed to identify all studies presenting longitudinal data on the effect of ANTH-BC on either AD and/or central PWV in cancer patients of all ages. An inverse-variance weighted random-effect model was performed with differences from before to after chemotherapy, as well as for short vs. mid-term effects.

**Results:**

Of 2,130 articles identified, 9 observational studies with a total of 535 patients (mean age 52 ± 11; 73% women) were included, of which four studies measured AD and seven PWV. Short-term (2–4 months), there was a clinically meaningful increase in arterial stiffness, namely an increase in PWV of 2.05 m/s (95% CI 0.68–3.43) and a decrease in AD (albeit non-significant) of −1.49 mmHg-1 (−3.25 to 0.27) but a smaller effect was observed mid-term (6–12 months) for PWV of 0.88 m/s (−0.25 to 2.02) and AD of −0.37 mmHg-1 (−1.13 to 0.39). There was considerable heterogeneity among the studies.

**Conclusions:**

Results from this analysis suggest that in the short-term, ANTH-BC increases arterial stiffness, but that these changes may partly be reversible after therapy termination. Future studies need to elucidate the long-term consequences of ANTH-BC on arterial stiffness, by performing repeated follow-up measurements after ANTH-BC termination.

**Systematic Review Registration:**

[www.crd.york.ac.uk/prospero/], identifier [CRD42019141837].

## Highlights

– Besides myocardial dysfunction, vascular toxicity has been recognized as a potential side effect of ANTH-BC that can be quantified by measurement of arterial stiffness, a robust surrogate marker of cardiovascular disease.
– Results from this analysis suggest that in the short-term, ANTH-BC increases arterial stiffness, but that these changes may (partly) be reversible after therapy termination.
– This is a novel finding and different from the permanent negative effects of ANTH-BC on myocardial function.
– However, given the high heterogeneity among studies included in this meta-analysis, additional studies will have to address the limitations, including measurement of confounders, and performing repeated and standardized follow-up measurements of arterial stiffness after ANTH-BC termination.
– Assessment of arterial stiffness may have the potential to contribute to risk prediction and clinical decision making in patients with ANTH-BC.

## Introduction

Heart disease and cancer are the leading causes of mortality worldwide (1). Due to remarkable improvements in screening, diagnosis, and treatment of many cancers, the number of cancer survivors is steadily increasing (2). However, cancer survivors have an increased risk for cardiovascular disease (CVD), either as a result from shared cardiovascular risk factors and suboptimal lifestyle choices or from toxicities of cancer treatment (3–5). A retrospective cohort study has shown that 10 years after cancer diagnosis the risk for death from CVD exceeds the risk of death from cancer (3).

Anthracyclines are very effective chemotherapeutic agents used for treatment of solid tumors and hematologic malignancies. However, due to their dose-dependent cardiotoxic effects, such as systolic and/or diastolic left ventricular (LV) dysfunction and heart failure (6–10), their repetitive administration is limited. Hence, monitoring of LV function by echocardiography before and after treatment is recommended (11, 12). Additionally, many anticancer drugs also have adverse effects on the vascular endothelium (13, 14), It has been proposed that anthracycline-based chemotherapy (ANTH-BC) may increase arterial stiffness (15) *via* generating reactive oxygen species and promoting oxidative stress (16, 17). This in turn leads to structural changes within the vascular matrix and thus interferes with the regulation of vascular smooth muscle tone (14). Both, *in vitro* and *in vivo* studies found that ANTH-BC also causes apoptosis of vascular endothelial cells, which may impair vasodilatory and contractile responses and lead to endothelial dysfunction (18, 19).

The most established non-invasive methods to assess central arterial stiffness are central pulse wave velocity (PWV) (20) and aortic distensibility (AD) by cardiac magnetic resonance (CMR) or echocardiography (20, 21). Both methods have been shown to predict CV events and CV mortality in various populations (22, 23).

Previous studies on the vasculotoxic effects of chemotherapies have mainly focused on anti-angiogenic drugs and some of the newer anticancer signaling inhibitors (24, 25). A recent review and meta-analysis has summarized effects of various vasculotoxic chemotherapies, including anthracyclines, on arterial stiffness from longitudinal and cross-sectional studies (26). Due to often various successive treatments in cancer patients, these cross-sectional studies do not allow the identification of the vasculotoxic effect of isolated ANTH-BC. ANTH-BC–induced vasculotoxicity may further be aggravated by the individual CV risk factor profile (i.e., current smoking, obesity, etc.), which are difficult to fully control for in cross-sectional studies. To date, several small longitudinal studies have assessed arterial stiffness before and after ANTH-BC, but the vascular effects of ANTH-BC over time remain unclear. An evidence synthesis is important because long-term vascular dysfunction may increase the risk for cardiovascular events and mortality (22, 27–29). Therefore, we have conducted a systematic review to appraise the literature regarding the time-dependent effect of ANTH-BC on markers of central aortic stiffness, namely PWV and AD measured before and after ANTH-BC in cancer patients.

## Methods

### Study Design

The search was conducted according to the preferred reporting items for systematic reviews and meta-analyses (PRISMA) recommendations. The original study protocol was registered prospectively in PROSPERO (CRD42019141837).

### Study Eligibility

Studies were eligible if they met all of the following criteria: (a) experimental or observational studies (prospective or retrospective); (b) reporting on the effect of ANTH-BC on either AD and/or central [carotid-femoral (cf)/aortic arch/carotid artery] PWV in cancer patients of all ages; (c) longitudinal assessment with baseline measurement before administration of anthracyclines and at least one measurement during or after ANTH-BC; (d) based on human data. We did not include studies which provided PWV from peripheral arteries or derived from pulse wave analysis, due to the fact that PWV is not directly measured in pulse wave analysis but calculated based on the estimated distance of assumed reflection sites (30).

### Database Search

The MEDLINE, Embase, Web of Science and the Cochrane Library databases were searched for eligible studies from database inception to February 18, 2021. The search strategy was built based on the PICO strategy. A combination of free textwords and MeSH subheadings were used, including the terms *cardiotoxicity, aortic distensibility, central pulse wave velocity*, *anthracycline*, *doxorubicin*, *daunorubicin*, *adriamycin*, *idarubicin*, *epirubicin*, appropriately linked with the Boolean operators AND or OR. Case reports, comments, and editorials were excluded. No language restrictions were applied. The full search algorithm for each database can be found in the Supplementary Appendix (Supplementary Table 1).

### Study Selection and Data Extraction

Upon removal of duplicate publications, the title and abstract of the selected studies were screened by 3 independent reviewers (C.S., P.E. N.G.). For each potentially eligible study, two reviewers (C.S., P.E.) independently assessed the full manuscripts. In cases of disagreement, a decision was made by consensus or the third reviewer was consulted. The reference lists of selected publications were also manually searched to identify additional eligible studies. For data extraction, a template was used including information on study size and design, baseline population, location, age at baseline, anthracycline-dose, duration of follow-up, type of outcome assessment, type and numbers of outcomes, concomitant treatment, comorbidities of population and the reported degree of adjustment.

### Risk of Bias Assessment

Risk of bias was assessed using the validated National Institute of Health (NIH) assessment tool for Before-After (Pre-Post) studies without control group (31). Cut-offs were used to judge overall risk of bias with 8–12 points indicating low risk, 5–7 points indicating moderate and 1–4 indicating high risk of bias. In addition, we used the Grading of Recommendations Assessment, Development and Evaluation (GRADE) method to assess the quality of evidence in the current systematic review (32). The GRADE method evaluates each outcome separately based on the quality of evidence (including the risk of bias, study design, consistency and directness of findings) and further considers the magnitude of effect. The evidence is categorized as either high, moderate, low or very low.

### Statistical Analysis

Mean differences were calculated from the differences between group means at different time points. Standard deviations (SD) of the mean differences (MD) were derived by using reported *p*-values from repeated measure analyses using the following formula SD = MD × √(n)/t (33), with n being the number of patients, and t the t-value for the given *p*-value and degrees of freedom according to the table on critical values of the Student’s t distribution.

Measurement units were converted where appropriate. An inverse variance weighted random-effect model was used to obtain the pooled mean difference with 95% CI for the change in outcome from before to after ANTH-BC treatment, separated by time-point of assessment into short-term (2–4 months) and mid-term effects (6–12 months).

We constructed forest plots, and assessed heterogeneity using the I^2^ statistic, with I^2^ ≤ 25% considered low, 25% < I^2^ < 75% moderate, and I^2^ ≥ 75% high (34).

Sensitivity analyses were performed to assess the impact of age (< / ≥ 50 years), cumulative ANTH-BC dose (< / ≥ 200 mg/m^2^), and assessment method (CMR vs. echocardiography for AD and CMR vs. Doppler echography for PWV) on vasculotoxicity. Results of all studies (AD and PWV data) were collated by expressing the mean change relative to mean baseline. Dose-response relationship was assessed by linear regression between arterial stiffness ratio relative to baseline and cumulative mean dose (if only range of dose was indicated, the central value was used). Statistical analyses were performed using Rev Manager [Version 5.3, The Cochrane Collaboration] and R [Version 4.1.2, R Core Team].

## Results

### Study Selection and Characteristics

**FIGURE 1:**
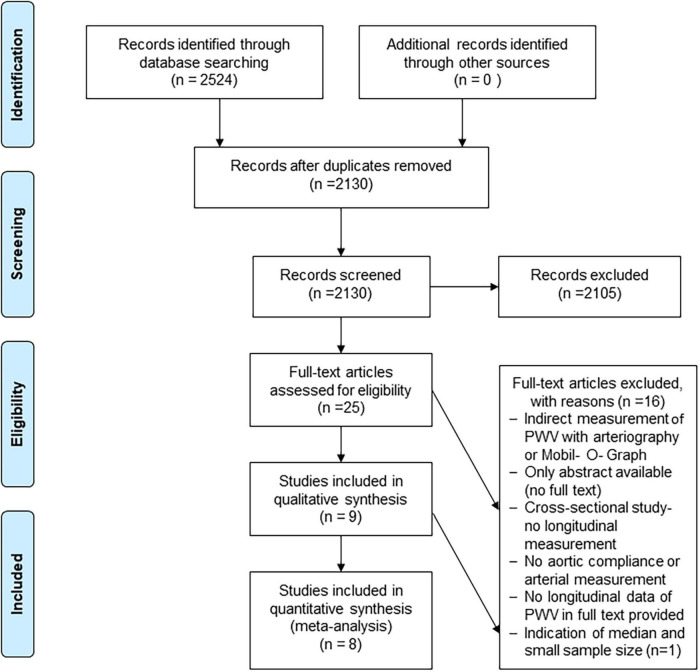
Flowchart for study inclusion, adapted from the PRISMA statement. Flow chart illustrating the study search, screening and selection processes.

Of the 2,130 studies identified, 9 studies met the inclusion criteria for this review (Figure 1), with clinical characteristics shown in Table 1.

**TABLE 1:** Description of the included studies.

Source	Cancer type (%)	Age	Cumulative dose (mg/m^2^)	Sample size n (% female)	Outcome assessment	Baseline PWV [m/s]	Follow-up PWV [m/s]	Baseline AD [mmHg^–1^]	Follow-up AD [mmHg^–1^]	CV risk factors
Novo et al. (43)	Breast cancer	56 ± 12	NA 4 cycles of Anthracycline treatment (every 21 days)	133 (100%)	Carotid arteries ultrasound	Median (IQR): 5.5 (5.15–6.4)	Median (IQR): 3 months: 6.7 (5.6–7.2) (*p* > 0.004) 6 months: 5.75 (5.2–6.7) (*p* > 0.05) 12 months: 5.7 (5.15–6.6) (*p* > 0.05)			Diabetes (13.5%) Hypertension (22%) Dyslipidemia (22%) Smoking (13.5%) Family history of CVD (18%) **Inclusion criteria**: LVEF > 50% Absence of: Coronary artery disease Hemodynamically relevant valvular heart disease Carotid atherosclerotic plaque **Exclusion criteria:** Pre-existing LV dysfunction before start of chemotherapy Severe liver or renal dysfunction
Mihalcea et al. (42)	Lymphoma (non-Hodgkin)	58 ± 11	8 ± 2 cycles of Doxorubicin at 50 mg/m^2^= 429 ± 61 after 3rd cycle: ∼150	110 (54%)	Echo right common carotid artery,	6.7 ± 1.1	3rd cycle 7.2 ± 1.2 (*p* < 0.05) Final 7.8 ± 1.5 (*p* < 0.05)			Diabetes (4%) Hypertension (17%) Dyslipidemia (8%) Smoking (9%) **Exclusion criteria:** History of CV disease History of radiotherapy
Turan et al. (43)	Lymphoma (non-Hodgkin)	52 (36–68)	6 cycles of Doxorubicin 436 ± 94	10 (80%)	SphygmoCor system (AtCor Medical, Sydney, Australia)	Median (min-max): 9.08 (8.12–9.76)	First cycle: Median (min-max) 10.31 (8.22–12.62) Sixth cycle 9.64 (8.22–12.62)			Hypertension (20%) Dyslipidemia, (20%) Smoking (10%) **Exclusion criteria**: History of coronary artery disease and heart failure
Chaosuwannakit et al. (35)	Breast cancer (48%) Lymphoma (28%) Leukemia (25%)	52 ± 11 (24–65)	Doxorubicin 215; 60–320 Daunorubicin 265; 100–600	Cancer: 40 (70%) Healthy controls: 13	CMR PC-CMR	6.9 ± 2.3	3.6 months: 13.5 ± 4.7 (*p* < 0.0001)	4.1 ± 1.6	3.6 months: 1.9 ± 1.2 (*p* < 0.0001)	Diabetes (13%) Hypertension (33%) Hyperlipidemia (23%)
Grover et al. (38)	Breast cancer (100%)	54 ± 11	3–6 cycles of Epirubicin at 100 mg/m^2^ = 300–600 3–6 cycles of Doxorubicin at 50 mg/m^2^ = 150–300	27 (100%) ANTH-BC: 15 TZM: 12 Healthy: 12	CMR PC-CMR	6.8 ± 3.2	1 month: 7.8 ± 4.3 (*p* > 0.05) 4 months: 8.9 ± 6.4 (*p* < 0.05) 12 months: 8.2 ± 4.2 (*p* < 0.05)	Anth-group only: 9.2 ± 2.8 All patients 8.1 ± 3.6	All patients, 4 months: 5.7 ± 3.2 (*p* < 0.001) 12 months: 6.9 ± 2.3 (*p* > 0.05) Anth-group only, 12 months: 6.8 ± 2.5 (p = 0.009)	Diabetes (15%) Hypertension (19%) Hypercholesterolemia (37%) Smokers: current (7%) Smokers: ex/41%) Family history of CAD (26%)
Drafts et al. (36)	Breast cancer (42%) Lymphoma (32%) Leukemia (24%) Myelodysplastic syndrome (2%)	50 ± 2 (19- 80)	Doxorubicin in 37 patients: 240; 50- 375 Daunorubicin in 16 patients: 180; 26–500	53 (58%)	PC-CMR	6.7 ± 0.5	6 months: 10.1 ± 1 (*p* = 0.0006)			Diabetes (13%) Hypertension (40%) Hyperlipidemia (25%) Smoking (45%) Coronary artery disease (8%)
Mizia- Stec et al. (37)	Breast cancer (100%)	50 ± 9 (35–68)	Doxorubicin: 278 ± 55; 100–300 Epirubicin: 414; 150–630	31 (100%)	Echo	16.7 ± 11.8	9–12 months: 14.9 ± 8.4 (*p* > 0.05)			Controlled hypertension: 52% **Exclusion criteria:** -Heart failure -Uncontrolled hypertension -Diabetes -CAD -Left side chest wall radiation -Currently smoking
Daskalaki et al. (41)	Lymphoma Non-Hodgkin 45 (62%) Hodgkin 25 (386%)	44 ± 19 Non- Hodgkin 52 ± 17 Hodgkin 28 ± 9	Doxorubicin 3 months: 150–200 End of treatment: 300–400	70 (47%)	Echo			3.31 ± 0.27 (2.48 ± 0.2 10^–6^ × dyn^–1^ × cm^2^)	3 months: 3.21 ± 0.24 (*p* = 0.059) *(2.41* ± *0.18*^†^ *10*^–6^ × *dyn*^–1^ × *cm*^2^) End of treatment: 3.15 ± 0.31 (*p* < 0.0001) *(2.36* ± *0.23*^‡^ *10*^–6^ × *dyn*^–1^ × *cm*^2^	Currently smoking: 11% **Exclusion criteria:** -History of myocardial infarction -Heart failure -Diabetes mellitus -Renal failure -Treatment with beta blockers, ARBs or ACE inhibitors
Jordan et al.* (39)	ANTH-BC: Breast cancer (44%) Leukemia (18%) Lymphoma (31%) Sarcoma (7%)	51 ± 12	Doxorubicin: 232 ± 103	ANTH-BC: 61 (69%) Non-ANTH-BC*:15 Healthy: 24	PC-CMR			1.68 ± 1.31	6 months: 1.98 ± 1.70 (*p* = 0.28)	ANTH-BC patients: Diabetes: 18% Hypertension: 38% Hyperlipidemia 26% Known CAD: 5%

*Non ANTH-BC group: breast cancer patients treated with trastuzumab regimen with either Docetaxel or Taxol (n=13) and patients treated for a hematologic malignancy with either all Transretinoic acid (n=1) or Bendamustine/Rituxan therapy (n=1) ACE, angiotensin-converting–enzyme; AD, aortic distensibility; ANTH-BC, anthracycline-based chemotherapy; ARBs, angiotensin-receptor blockers; CAD, coronary artery disease; CV, cardiovascular PC-CMR, phase-contrast cardiovascular magnetic resonance; PWV, pulse- wave- velocity; TZM, trastuzumab.

**FIGURE 2:**
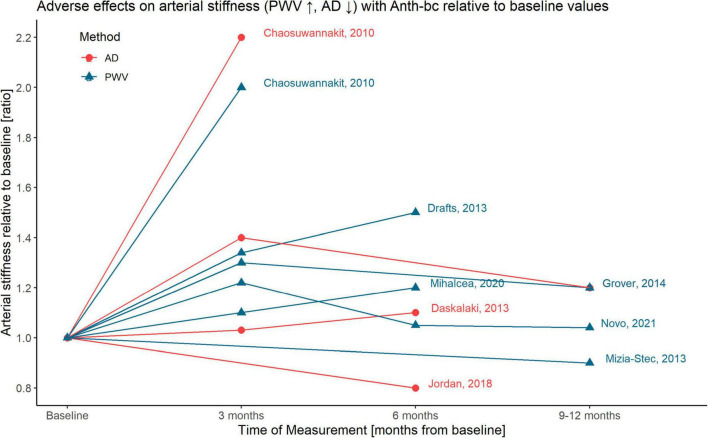
Graphical illustration of time-dependent adverse effects on arterial stiffness with ANTH-BC relative to baseline values; AD, aortic distensibility; PWV, pulse-wave-velocity.

All studies were published between 2010 and 2021 and included patients with solid tumors, such as breast cancer or sarcoma (35–40), or hematologic malignancies, such as lymphoma and leukemia (35, 36, 39, 41–43), or a combination thereof (35, 36, 39). All studies were prospective with data provided from before treatment as well as after a follow-up period between 1 and 14 months (Figure 2). Three studies included a control group consisting of healthy, age-matched volunteers (35, 38), or a cancer group without ANTH-BC (39). Studies were based on 10–133 patients, with mean age 52 (SD 11) years, and 73% women. Two of the studies included in this analysis excluded patients with CV comorbidities (37, 41). Concomitant treatments mostly included cyclophosphamide, trastuzumab, taxanes and/or radiotherapy. Based on available data, we decided to perform meta-analyses on short-term effects at 2–4 months, which coincided with conclusion of ANTH-BC in breast cancer and some lymphoma patients, and at 6–12 months, at which time point also all lymphoma patients had concluded their treatment (44, 45), while some patients were likely to have terminated ANTH-BC several months previously. Mean cumulative dose of Doxorubicin delivered was 310 mg/m^2^ (range 215–436 mg/m^2^, range for individual patients 50–436 mg/m^2^). Dose-response relationship showed a non-significant regression between arterial stiffness ratio and administered ANTH-BC-dose (*r* = 0.06, *p* = 0.594). Other anthracyclines included were Daunorubicin and Epirubicin, which have comparable or lower cardiotoxic effects compared to Doxorubicin (12, 46).

Four studies provided data on AD and seven on PWV (AD and PWV were concomitantly reported in two studies, Figure 2). Three studies measured AD by cardiovascular magnetic resonance imaging (CMR) (35, 38, 39) and one by echocardiography (37, 41). PWV was assessed using CMR (35, 36, 38), echocardiography (37, 42), SphygmoCor (43) or carotid artery ultrasound (40) and was reported in m/s by all studies.

### Risk of Bias Analysis and Quality of Evidence

Risk of bias was moderate in most included studies (5–8 points, Table 2). Only four studies provided sufficient information on eligibility criteria (37, 38, 41). None of the nine included studies provided a study flow. Results of PVW and AD were of very low certainty. The evidence is based solely on observational studies and despite good generalizability regarding the study population and each outcome, we found some unexplained heterogeneity. Due to the small number of studies, publication bias was not assessed. Imprecision, inconsistency and risk of bias were a serious concern for both outcomes. Supplementary Table 1 summarizes the assessment of evidence quality.

**TABLE 2:** Quality assessment of included studies using the NIH.

Criteria	Chaosu-wannakit	Drafts	Grover	Jordan	Daska-laki	Mizia-Stec	Mihalcea	Novo
1. Was the study question or objective clearly stated?	Yes	Yes	Yes	Yes	Yes	Yes	Yes	Yes
2. Were eligibility/selection criteria for the study population prespecified and clearly described?	No	No	Yes	No	Yes	Yes	Yes	Yes
3. Were the participants in the study representative of those who would be eligible for the test/service/intervention in the general or clinical population of interest?	Yes	Yes	Yes	Yes	No	No	Yes	Yes
4. Were all eligible participants that met the prespecified entry criteria enrolled?	n.r.	n.r.	n.r.	n.r.	n.r.	n.r.	n.r.	n.r.
5. Was the sample size sufficiently large to provide confidence in the findings?	Yes	Yes	Yes	Yes	Yes	Yes	Yes	Yes
6. Was the intervention (ANTH-BC) clearly described and delivered consistently across the study population?	Yes	Yes	Yes	Yes	n.r.	Yes	Yes	n.r.
7. Were the outcome measures prespecified, clearly defined, valid, reliable, and assessed consistently across all study participants?	Yes	Yes	Yes	Yes	Yes	Yes	Yes	Yes
8. Were the people assessing the outcomes blinded to the participants’ exposures/interventions?	Yes	Yes	n.r.	Yes	Yes	n.r.	n.r.	n.r.
9. Was the loss to follow-up after baseline 20% or less? Were those lost to follow-up accounted for in the analysis?	n.r.	n.r.	Yes	n.r.	Yes	Yes	No (loss more than 20%, baseline: 147, final assessment 110)	n.r.
10. Did the statistical methods examine changes in outcome measures from before to after the intervention? Were statistical tests done that provided p values for the pre-to-post changes?	Yes, but method n.r.	Yes	Yes	Yes	Yes	Yes	Yes	Yes
11. Were outcome measures of interest taken multiple times before the intervention and multiple times after the intervention (i.e., did they use an interrupted time-series design)?	No	No	No	No	No	No	No (once before intervention, but twice after (3rd and last cycle)	No
12. If the intervention was conducted at a group level (e.g., a whole hospital, a community, etc.) did the statistical analysis take into account the use of individual-level data to determine effects at the group level?	NA	NA	NA	NA	NA	NA	NA	NA
Overall rating	7/12	7/12	8/12	7/12	7/12	7/12	7/12	6/12

*Quality assessment tool for before-after (pre-post) studies with no control group.*

*The colours represent the quality of the studies included in this meta-analysis with red for high risk, yellow for uncertain and green for low risk of bias.*

### Aortic Distensibility

**FIGURE 3:**
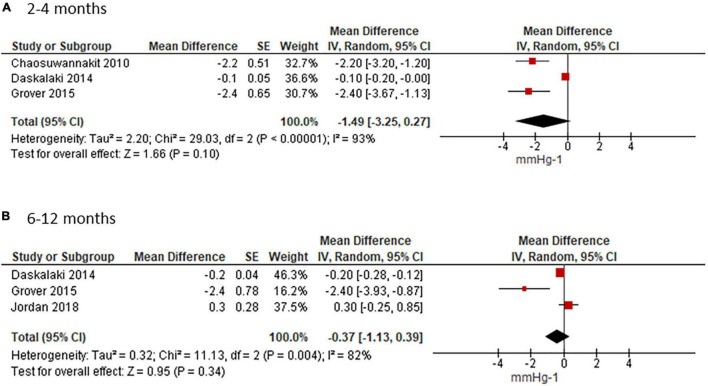
Effect of ANTH-BC on AD. Forest plots illustrating the effect of ANTH-BC on AD divided by time-point of assessment into **(A)** short-term (3–4 months) and **(B)** mid-term (6–12 months) effects.

Meta-analyses for short- and mid-term reporting on AD are summarized in Figure 3. AD was reported in mmHg^–1^ in all except one study (41), which we converted as 1 dyne/cm^2^ = 0.00075 mmHg.

Short-term analysis of studies assessing AD after 3 or 4 months, coinciding with termination of ANTH-BC in breast cancer and lymphoma patients who had 4 and 6 chemotherapy cycles, showed an effect of −1.49 mmHg^–1^ (95%CI −3.25; 0.27). There was considerable heterogeneity amongst these studies (Chi^2^ = 29.03, df = 2, *p* < 0.00001, I^2^ = 93%, Figure 3A). In the subgroup analysis for measuring method, heterogeneity disappeared in the CMR studies where AD was reduced significantly by −2.28 mmHg^–1^ (95%CI −3.06; −1.49, I^2^ = 0%) (Supplementary Figure 1A). This sub-group analysis corresponded to the sensitivity analysis for age and anthracycline dose since the study which used echocardiography was also the study with younger mean age (44 ± 19 years) and lower anthracycline dose (< 200 mg/m^2^) (41).

The mean weighted change in AD for the studies with follow-up at 6–12 months was −0.37 mmHg^–1^ (95% CI −1.13; 0.39, I^2^ = 82%, Figure 3B). Heterogeneity persisted in the subgroup analysis for assessment method in CMR studies (−0.95 mmHg^–1^; 95%CI −3.59; 1.69, I^2^ = 91%, Supplementary Figure 1B).

### Pulse Wave Velocity

Four studies reporting on PWV presented means with standard deviations (SD) at each time point, whereas two studies presented the median (40, 43). Since the study by Turan et al. (43) reported the median (range) it was included in the systematic review only (43). In the study by Novo and colleagues, PWV was indicated as median (interquartile range) (40). Due to the relatively large sample size of this study (*n* = 133), we included it into our meta-analysis by using the median as mean and approximated the standard deviation according to the following Cochrane formula: width of the interquartile range = 1.35 standard deviations (33). Two studies did not provide an exact *p*-value for PWV change but only indicated that it was non-significant (37, 40). Since the SD of the change could not be calculated for these studies, it was approximated by taking the mean SD of the other three studies who provided exact *p*-values. Another study stated that p was < 0.05. Using a conservative estimation, we calculate the SD of the mean change based on *p* = 0.049 (42). In a third study, PWV data at 4 months was only provided in a graph from which data was estimated visually (36).

**FIGURE 4:**
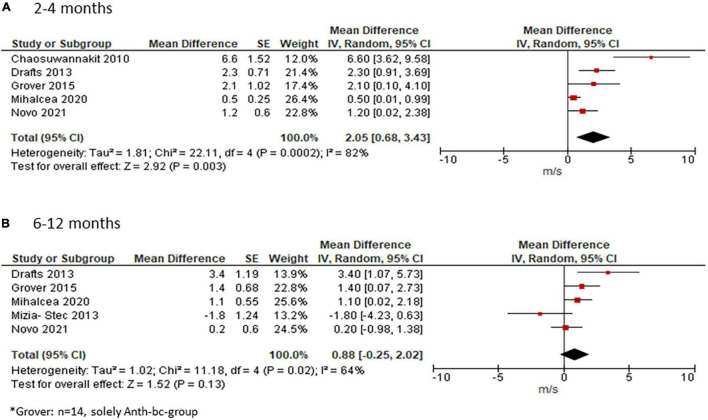
Effect of ANTH-BC on PWV. Forest plot illustrating the effect of ANTH-BC on PWV divided by time-point of assessment into **(A)** short-term (2–4 months, corresponding to subgroup analysis for CMR) and **(B)** mid-term (6–12 months) effects.

Meta-analysis of the five studies who provided data at 2–4 months showed an increase in PWV of 2.05 m/s (95%CI 0.0.68; 3.43) from before to after ANTH-BC (Figure 4A) with considerable heterogeneity among the studies (Chi^2^ = 21.89, df = 3, *p* < 0.0001, I^2^ = 82%). Subgroup analysis for CMR-studies only showed an increase in PWV of 3.34 m/s (95%CI 1.10; 5.58, Supplementary Figure 2A) with considerable heterogeneity amongst the studies (Chi^2^ = 7.22, df = 2, *p* = 0.03, I^2^ = 72%).

For studies with follow-up at 6–12 months, mean weighted change in PWV was 0.88 m/s (95% CI −0.25; 2.02, I^2^ = 64%, Figure 4B). Subgroup analysis for assessment method showed a significant effect of 2.16 m/s (95% CI 0.26; 4.07) in CMR studies with reduced heterogeneity (I^2^ = 53%, Supplementary Figure 2B).

## Discussion

This systematic review summarized the current evidence of the time-dependent effect of ANTH-BC on central aortic stiffness, assessed as AD or central PWV. Results from this meta-analysis suggest that in the short term (at termination of ANTH-BC), moderate dose ANTH-BC has a clinically meaningful effect on increasing arterial stiffness, presenting as an increase in PWV and a decrease in AD, albeit non-significant for AD. Findings from this study are in line with the results of a recent meta-analysis on this topic (26). However, as a novel finding, we observed smaller effects when measurements were performed at 6–12 months (Figure 2), suggesting at least partial recovery, which was supported by two out of the three studies who provided repeat measurements at short- and mid-term time points. This suggest that ANTH-BC vascular toxicity may at least in part be reversible, in contrast to myocardial toxicity. The risk of bias of the included studies was moderate. The quality of the studies included in this review was limited mainly by study design and methodology.

### Comparison With Other Studies

Over the past 10–15 years, an extensive body of literature has been published identifying increased arterial stiffness as a predictor of cardiovascular events and mortality.(22, 23, 47) AD has been found a sensitive parameter of arterial stiffness in patients younger than 50 years, while PWV is the more sensitive parameter after the age of 50.(21) According to a meta-analysis of general population studies, a 1 m/s increase in PWV, as found in our study in the long-term, corresponds to an age-, sex-, and risk factor-adjusted risk increase of approximately 14% in total CV events, CV mortality, and all-cause mortality,(48) underlining the clinical importance of this finding. According to a study by Redheuil et al. who assessed the predictive value of AD for mortality, hard CV events and HF events in 3,675 patients without clinical CVD (mean age 61 ± 10 years) (23), patients included in our meta-analysis had either a not elevated (35, 39) to twofold increased risk (38, 41) for CV events.

Our meta-analysis suggests that adverse effects of ANTH-BC on arterial stiffness may partially be reversible after ANTH-BC termination. Of the five studies that performed two follow-up measurements, one at 3 months and one at 6 months (36, 40–42) or 12 months (38, 40), three studies found a further worsening (36, 41, 42), while the study by Grover and Novo and colleagues found a recovery toward baseline values (Figure 2). Even though arterial stiffness parameters may partially recover from acute ANTH-BC exposure, this may not mean that long-term vasculotoxic effects will not be present. Nevertheless, at 5 or 10 years after treatment termination it will be difficult to ascribe increased arterial stiffness to certain chemotherapies, as other treatments, advanced age, cancer itself, or cardiovascular risk factors are known to also play a role. The largest study that was included in this meta-analysis showed a clear recovery of arterial stiffness after the initial decline at 3 months (40). Since both follow-up measurements at 6 and 12 months showed values equal to pre-anthracycline measurements despite further treatment with other chemo- or radiotherapies, this study added considerably to the conclusion that the adverse effect of ANTH-BC to arterial stiffness may be reversible. The hypothesis of partial recovery of adverse effects over time will need to be confirmed in longitudinal studies which measure before, at completion of ANTH-BC and at a later follow-up time. Further, it is clinically important to assess whether partial recovery may be due to cardioprotective treatment of diagnosed cardiotoxic side-effects following cancer therapy.

In our meta-analysis, baseline AD values of three studies were within the range of 1.7 ± 1.3 to 4.1 ± 1.6 mmHg^–1^ (35, 39, 41), and in the range of reference values in the literature for age-matched, healthy individuals (3.1 ± 1.8 to 4.0 ± 1.6 mmHg^–1^) (49). However, baseline AD in the study by Grover et al. was markedly higher (8.1 ± 3.6 mmHg^–1^). Similarly, values for baseline PWV from the study by Mizia-Stec and colleagues, who measured cfPWV by Doppler echography were noticeably higher (16.7 ± 11.8 m/s) compared to those assessed in the other studies (6.7 ± 0.5 to 6.9 ± 2.3 m/s), which measured aortic arch PWV by CMR (35, 36, 38). Surface cfPWV has been found to overestimate true aortic PWV by 2–3 m/s (21), however, this methodological difference cannot explain the almost 10 m/s higher values. However, the unusually high SD of 11 m/s in the study by Mizia-Stec and colleagues raises some doubt about the reliability of their PWV data.

### Sources of Heterogeneity

Overall, we found high heterogeneity amongst the studies included in the random-effect analyses for AD and PWV that persisted when performing sensitivity and subgroup analyses. Possible reason for the observed heterogeneity could be the clinical diversity of the study populations with various degrees of cardiovascular risk, bias from patient drop-out, or lack of blinding. None of the studies could be found in a trial registry for verification of reported results with study protocol, and none presented a patient flow. In addition, publication bias may be present.

### Potential Modulators of Vasculotoxicity

Vasculotoxicity is likely to be modulated by age, the effect of cumulative ANTH-BC dose, the individual cardiovascular risk factor profile, additional chemo- and radiotherapies, and cardioprotective medication. It is well established that cumulative ANTH-BC dose plays an important role in the development of cardiotoxicity (12). While Chaosuwannakit et al. found an association between cumulative ANTH-BC dose and worsening of AD (*r* = 0.34, *p* = 0.02), Drafts et al. could not confirm these findings (*p* = 0.6). In this meta-analysis, studies with moderate ANTH-BC-dose (between 200 and 450 mg/m^2^) show either a much (ratio of 2.2) or somewhat increased arterial stiffness (ratio of 1.1–1.4) or a decrease (ratio of 0.8–1.0, see Figure 2) leading to non-significant regression (*r* = 0.06, *p* = 0.594). However, this may not be interpreted as a non-existing dose-response relationship but rather be a consequence of the large heterogeneity between the included studies.

Most of our studies investigated the relation between blood pressure and vascular injury (35–38, 41). Grover et al. found a higher increase in arterial stiffness in patients with higher systolic BP. A higher PWV at baseline and greater increase over time with higher systolic BP was also found by Drafts et al., and Daskalaki et al. found decreased AD to be associated with higher systolic BP. Contrarily, Mizia-Stec and colleagues did not find any relationship between the diagnosis of systemic hypertension and ANTH-BC induced changes in PWV. However, none of the studies adjusted changes in arterial stiffness for changes in BP, which has a direct impact on PWV (50). As blood pressure tends to be decreased with ANTH-BC (51), the increase in arterial stiffness measured by PWV found in this and the previous meta-analysis (26) may be underestimated (50).

None of our studies found a significant effect of additional chemotherapies (35, 36), however, the small sample sizes may have precluded the detection of such associations. Future studies are warranted to gain more insight into the effect of age, cumulative ANTH-BC dose, the presence of cardiovascular risk factors and the addition of co-medication on vascular function.

### Strengths and Limitations

Subgroup analyses of different time points has allowed the detection of a potential (partial) reversibility of adverse effects by ANTH-BC on arterial stiffness. Another strength of this meta-analysis is the inclusion of studies assessing central arterial stiffness only. This is important since central (i.e., aorta and carotid arteries) and peripheral (i.e., brachial or femoral) arteries differ in their passive and active contractile properties (52). In contrast to a recent meta-analysis on the same topic, using *p*-values of repeat measure analyses provided us with a higher power to detect significant results due to a more efficient adjustment for confounders. GRADE assessment allowed an in-depth rating of the evidence for each outcome.

A limitation of our study was that all included studies were observational and expectedly did not include a truly comparable control group of cancer patients. This greatly limits the value of a meta-analysis (33). Therefore, the effect of cancer itself, presence of CV risk factors or other confounding treatments and comorbidities could not be identified. Secondly, except for two studies (40, 42), they were based on small numbers of participants, which explains the large CIs of some of the studies. Another limitation of this meta-analysis is that the assumption of a normal distribution has been made for PWV in the study by Novo et al., (40) even though data was indicated as median (IQR). Studies did not report ANTH-BC duration, making it difficult to estimate the follow-up time after ANTH-BC termination for the various cancer patients. Unfortunately, none of included studies were able to provide individual patient data.

### Conclusions and Clinical Implications

Results from this analysis suggest that in the short-term, ANTH-BC increases arterial stiffness, but that these changes may (partly) be reversible after therapy termination. Future studies need to elucidate the long-term consequences of ANTH-BC on arterial stiffness, by performing repeated and standardized follow-up measurements after ANTH-BC termination to confirm or challenge the findings of reversibility of arterial stiffness put forward by the study of Novo and colleagues. Reporting of data needs to be improved and availability of individual patient data in repositories is highly desirable. The adverse effect of ANTH-BC on arterial stiffness likely applies to the whole vasculature and expands beyond the myocardium. Several reviews highlighted the importance of arterial stiffness in the prediction of all-cause cardiovascular outcomes (22, 27–29). Therefore, non-invasive assessment of arterial stiffness may be used for detection of early cardiovascular injury in asymptomatic patients at risk during treatment and effects of cardio-/vasculo-protective treatments.

## Data Availability Statement

The original contributions presented in this study are included in the article/Supplementary Material, further inquiries can be directed to the corresponding author.

## Author Contributions

CS, PE, and MW were involved in the conception and design. CS, TM, and PE performed the analysis and interpretation of this meta-analysis and drafted the manuscript. NG-J assisted with screening of potential studies and was further involved in the design of this analysis. AB was involved in the analysis and interpretation of data and revised the manuscript. KC and TS revised the manuscript critically to provide intellectual content. All authors contributed to the article and approved the submitted version.

## Conflict of Interest

The authors declare that the research was conducted in the absence of any commercial or financial relationships that could be construed as a potential conflict of interest.

## Publisher’s Note

All claims expressed in this article are solely those of the authors and do not necessarily represent those of their affiliated organizations, or those of the publisher, the editors and the reviewers. Any product that may be evaluated in this article, or claim that may be made by its manufacturer, is not guaranteed or endorsed by the publisher.

## Abbreviations

AD: aortic distensibility
ANTH-BC: anthracycline-based chemotherapy
CFPWV: carotid-femoral pulse-wave velocity
CI: confidence interval
CMR: cardiac magnetic resonance
PR: phase- contrast
PWV: pulse-wave-velocity
CVD: cardiovascular disease
CV: cardiovascular.
