# Supplementary material for: Time-Dependent Effect of Anthracycline-Based Chemotherapy on Central Arterial Stiffness: A Systematic Review and Meta-Analysis
Source: Front Cardiovasc Med. 2022 Jul 5;9:873898. doi: 10.3389/fcvm.2022.873898 (PMC9295862; doi:10.3389/fcvm.2022.873898)
Supplement: Supplementary file 1 [file Data_Sheet_1.DOCX]

Supplementary data

Supplement Figure 1: Subgroup analysis for the association of Anthracycline-based chemotherapy on aortic distensibility, CMR-studies only

1. 2-4 months


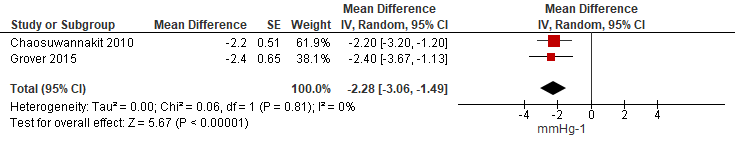


1. 6-12 months


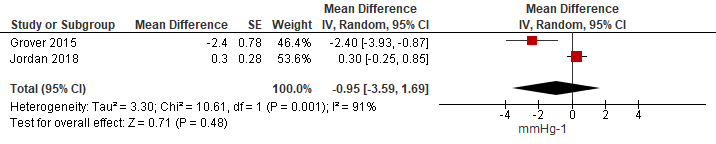


Supplement Figure 2: Subgroup analysis for the association of Anthracycline-based chemotherapy on pulse-wave-velocity, CMR-studies only

1. 2-4months


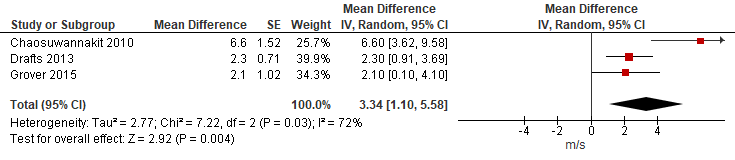


1. 6-12 months


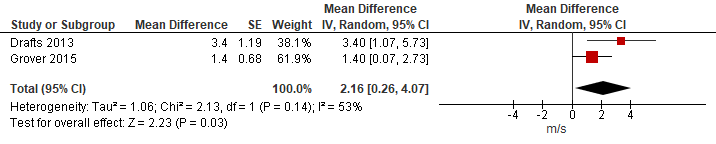


Supplement Figure 3: Sensitivity analysis for short-term effects of Anthracycline-based chemotherapy on aortic distensibility and pulse-wave-velocity after removing the study by Chaosuwannakit et al. 2010

1. Aortic distensibility, 2-4months


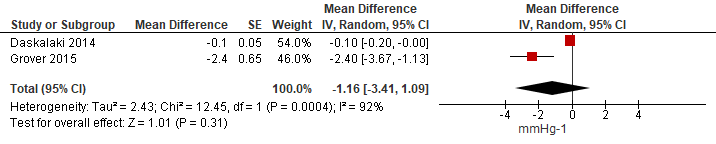


1. Pulse-wave-velocity, 2-4 months


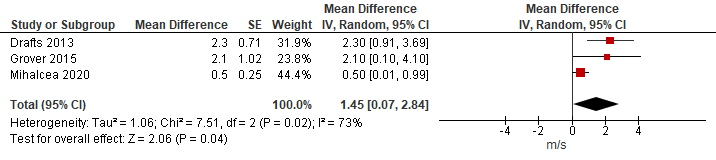


Supplement Table1: The effect of anthracycline- based chemotherapy (Anth- bc) on arterial stiffness measured through aortic distensibility and pulse- wave velocity

| **Quality assessment** | | | | | | | | **No of study**  **subjects** | **Mean Difference (95% CI)** | **Quality of Evidence (GRADE)** | **Importance** |
| --- | --- | --- | --- | --- | --- | --- | --- | --- | --- | --- | --- |
|  | **Design** | **No of studies** | **Risk of bias** | **Inconsistency** | **Indirectness** | **Imprecision** | **Other considerations** | **Total** |  |  |  |
| Outcome 1 |  |  |  |  |  |  |  |  |  |  |  |
| Aortic  Distensibility | Observational^1^ | 4 | -1^2^ | Serious concern^3^ | No concern^4^ | Serious concern^5^ | No concern^6^ | 198 | Short-term:  -1.49 mmHg^-1^ (95%CI -3.25; 0.27)  Mid-term:  -0.37 mmHg^-1^ (95% CI -1.13; 0.39) | Very low | Critical |
| Outcome 2 |  |  |  |  |  |  |  |  |  |  |  |
| Pulse-wave-velocity | Observational^7^ | 6 | -1^8^ | Serious concern^9^ | No concern^10^ | Serious concern^11^ | No concern^12^ | 394 | Short-term:  2.05 m/s  (95%CI 0.68; 3.43)  Mid-term:  1.09 m/s (95%CI -0.38; 2.56) | Very low | Critical |

Footnotes

**AD:**

^1^ One of the studies was a prospective cohort study and three studies were prospective case-control studies.

^2^Assessed by the NIH. Quality Assessment Tool for Before-After (Pre-Post) Studies With No Control Group. One study was of good quality and three of moderate quality; therefore bias is a concern in this outcome.

^3^We found high unexplained heterogeneity: short-term: I^2^= 93.2%, p<0.00001 (considerate heterogeneity), one study has no overlap of 95% CI with other studies; mid-term: I^2^= 82%, p= 0.004 (considerate heterogeneity), 95% CI barely overlap.

^4^Only one of the included studies in this analysis excluded patients with CV comorbidities and coronary artery disease. All the other studies included male and female cancer patients with CV comorbidities who typically receive Anth-bc. Therefore, we think that the results are representative for this patient group.

^5^Relatively small number of participants, 95% CI contains 0 and effect size is not statistically significant, therefore imprecision is a concern in this outcome.

^6^No evidence for publication bias. Due to low number of studies (n=4), publication bias was not formally assessed. No upgrade for a large magnitude of effect, only some studies accounted for confounders (cumulative Anth-bc dose, baseline blood pressure), however, other important confounders were not taken into account (residual confounding); no evidence for dose-response relationship.

**PWV:**

^7^Five of the studies were prospective cohort studies and two studies were prospective case-control studies.

^8^Assessed by the NIH. Quality Assessment Tool for Before-After (Pre-Post) Studies With No Control Group. One study was of good quality, four of moderate quality and one of low quality, therefore bias is a concern in this outcome.

^9^ We found high unexplained heterogeneity: short-term: I^2^= 82%, p<0.00001 (considerate heterogeneity), in one study the 95% CI barely overlaps with other studies; mid-term: I^2^= 68%, p= 0.02 (considerate heterogeneity), in one study the 95% CI barely overlaps with other studies.

^10^ Only one of the included studies in this analysis excluded patients with CV comorbidities. All the other studies included male and female cancer patients with CV comorbidities who typically receive Anth-bc. Therefore, we think that the results are representative for this patient group.

^11^Small number of participants. In the meta-analysis of Vlachopoulos et al. (2010), the threshold of 1m/s was defined as clinically relevant. Therefore, we used the threshold of 1m/s for our evaluation. Given that this threshold falls within the 95% CI, imprecision is a concern in this area.

^12^No evidence for publication bias, due to low number of studies (n=6), publication bias was not formally assessed. No upgrade for a large magnitude of effect, only some studies accounted for confounders (cumulative Anth-bc dose, blood pressure), however, other important confounders were not taken into account (residual confounding); no evidence for dose-response relationship.

References

Vlachopoulos C, Aznaouridis K, Stefanadis C. Prediction of Cardiovascular Events and All-Cause Mortality With Arterial Stiffness: A Systematic Review and Meta-Analysis. Journal of the American College of Cardiology. 2010;55(13):1318-27.
