# Supplementary material for: Time-Dependent Effect of Anthracycline-Based Chemotherapy on Central Arterial Stiffness: A Systematic Review and Meta-Analysis
Source: Front Cardiovasc Med. 2022 Jul 5;9:873898. doi: 10.3389/fcvm.2022.873898 (PMC9295862; doi:10.3389/fcvm.2022.873898)
Supplement: Supplementary file 2 [file Data_Sheet_2.DOCX]

**eAppendix
(all searches run 24.07.2019; Update 21.01.2020, 2^nd^ Update 18.02.2021, 3^rd^ Update 25.11.2021)**

**July 24, 2019 January 21, 2020 February 18, 2021**

| *Databases* | *Before deduplica-tion* | *After deduplica-tion* | *Before deduplica-tion* | *After deduplica-tion* | *Before deduplica-tion* | *After deduplica-tion* |
| --- | --- | --- | --- | --- | --- | --- |
| Medline Ovid | 658 | 658 | 678 | 677 | 703 | 703 |
| Embase Ovid | 1115 | 879 | 1158 | 920 | 1282 | 1019 |
| Cochrane Library | 26 | 24 | 28 | 27 | 30 | 27 |
| Web-of-Science | 79 | 23 | 84 | 25 | 94 | 25 |
| Google Scholar | *100 | 82 | *100 | 82 | *200 | 188 |
| ClinicalTrials.gov | 9 | 9 | 9 | 8 | 9 | 8 |
| WHO ICTRP | 21 | 21 | 72 | 72 | 59 | 59 |
| **Total** | **2008** | **1696** | **2129** | **1811** | **2377** | **2029** |

*in the meantime, the standard for Google Scholar retrieval is 200.

Date last searched: **November 25, 2021**

| *Databases* | *Before deduplica-tion* | *After deduplica-tion* |
| --- | --- | --- |
| Medline Ovid | 735 | 735 |
| Embase Ovid | 1383 | 1112 |
| Cochrane Library | 31 | 28 |
| Web-of-Science | 102 | 31 |
| Google Scholar | 200 | 154 |
| ClinicalTrials.gov | 11 | 10 |
| WHO ICTRP | 62 | 60 |
| **Total** | **2524** | **2130** |

**Total number of new results to screen after 3^rd^ update: 361**

3 blocks:
1) Neoplasms AND 2) Anthracyclines AND 3) Vascular Stiffness

Filters: no animal studies, no case reports/editorials/letters/conference abstracts etc.

**Ovid MEDLINE**(R) and Epub Ahead of Print, In-Process, In-Data-Review & Other Non-Indexed Citations, Daily and Versions(R) <1946 to November 24, 2021>

((exp Neoplasms/ or (cancer* or neoplasm* or carcinom* or carcinogen* or malignan* or tumo?r* or leukemi* or leukaemia* or AML or ALL or lymphom* or sarcom* or (multiple adj2 myelom*)).ti,ab,kw.) and (exp Anthracyclines/ or exp Antineoplastic agents/ or exp cardiotoxicity/ or (Anthracyclines or Anth-bC or Aclarubicin or Daunorubicin or Carubicin or Doxorubicin or Epirubicin or Idarubicin or Nogalamycin or Menogaril or Plicamycin or cardiotoxic* agent* or cardiotoxicit* or cardiac toxicit* or cardiovasc* toxicit* or card*-oncolog*).ti,ab,kw.) and (exp Aorta/ or (aortic adj1 (distens* or stiff* or elastic* or stretch* or compliance)).ti,ab,kw. or (exp Vascular stiffness/ or exp Elasticity or exp Pulse wave analysis/ or (aortic pulse? wave velocit* or aortic PWV or carotid PWV or carotid distensibil* or carotid- femoral pulse? wave velocit* or carotid- femoral PWV or central pulse? wave velocit* or central PWV or cfPWV).ti,ab,kw.) or ((Arterial or artery or vascular) adj3 (stiff* or elastic* or remodel*)).ti,ab,kw.)) not (exp animals/ not humans/) not (letter or news or comment or editorial or congress or case reports).pt

**Ovid Embase** <1974 to 2021 November 24>

(exp neoplasm/ or (cancer* or neoplasm* or carcinom* or carcinogen* or malignan* or tumo?r* or leukemi* or leukaemia* or AML or ALL or lymphom* or sarcom* or (multiple adj2 myelom*)).ti,ab,kw) AND (exp anthracycline/ or exp antineoplastic agent/ or exp cardiotoxicity/ or (Anthracyclines or Anth-bC or Aclarubicin or Daunorubicin or Carubicin or Doxorubicin or Epirubicin or Idarubicin or Nogalamycin or Menogaril or Plicamycin or cardiotoxic* agent* or cardiotoxicit* or cardiac toxicit* or cardiovasc* toxicit* or card*-oncolog*).ti,ab,kw) AND ((exp aorta/ or (aortic adj1 (distens* or stiff* or elastic* or stretch* or compliance)).ti,ab,kw) or (exp arterial stiffness/ or exp elasticity/ or exp pulse wave/ or (aortic pulse* wave velocit* or aortic PWV or carotid PWV or carotid distensibil* or carotid- femoral pulse* wave velocit* or carotid- femoral PWV or central pulse* wave velocit* or central PWV or cfPWV).ti,ab,kw) or ((Arterial or artery or vascular) adj3 (stiff* or elastic* or remodel*)).ti,ab,kw) not ((exp animal/ or nonhuman/) not exp human/) not (conference abstract or editorial or letter or note).pt not case report/

**Cochrane Database of Systematic Reviews**
Issue 11 of 12, November 2021
**Cochrane Central Register of Controlled Trials (CENTRAL)**
Issue 10 of 12, October 2021

(cancer* or neoplasm* or carcinom* or carcinogen* or malignan* or tumo*r* or leukemi* or leukaemia* or AML or ALL or lymphom* or sarcom* or (multiple NEAR/2 myelom*)) AND (Anthracyclines or Anth-bC or Aclarubicin or Daunorubicin or Carubicin or Doxorubicin or Epirubicin or Idarubicin or Nogalamycin or Menogaril or Plicamycin or cardiotoxic* agent* or cardiotoxicit* or cardiac toxicit* or cardiovasc* toxicit* or (card* NEXT oncolog*)) AND ((aortic NEAR/1 (distens* or stiff* or elastic* or stretch* or compliance)) OR ((arterial or artery or vascular) NEAR/3 (stiff* or elastic* or remodel*)) OR (aortic NEXT pulse? NEXT wave NEXT velocit*) OR "aortic PWV" OR "carotid PWV" OR (carotid NEXT distensibil*) OR (carotid-femoral NEXT puls? NEXT wave NEXT velocit*) OR (central NEXT pulse? NEXT wave NEXT velocit*) OR "central PWV" OR cfPWV)

**Web of Science** Core Collection

TS=((cancer* or neoplasm* or carcinom* or carcinogen* or malignan* or tumo*r* or leukemi* or leukaemia* or AML or ALL or lymphom* or sarcom* or multiple NEAR/2 myelom*) AND (Anthracyclines or "Antineoplastic Drugs" or Antineoplastics or "Anticancer Agents" or cisplatin* or cytotoxin* or chemotherapy or “anticarcinogenic agent*” or Anth-bC or Aclarubicin or Daunorubicin or Carubicin or Doxorubicin or Epirubicin or Idarubicin or Nogalamycin or Menogaril or Plicamycin or cardiotoxic* NEAR/2 agent* or cardiotoxicit* or cardiac NEAR/2 toxicit* or cardiovasc* NEAR/2 toxicit* or "card* oncolog*") AND ((aortic NEAR/2 (distens* or stiff* or elastic* or stretch* or compliance)) OR ((arterial or artery or vascular) NEAR/2 (stiff* or elastic* or remodel*)) OR "aortic pulse* wave velocity*" OR "aortic PWV" OR "carotid PWV" OR "carotid distensibil*" OR "carotid-femoral pulse* wave velocit*" OR "central pulse* wave velocit*" OR "central PWV" OR cfPWV)) NOT TS=((animal* OR plant* OR rats OR mice OR pigs) NOT (human* OR patient*)) *AND* **DOCUMENT TYPES:** (Article)

**Google Scholar**

anthracycline|"cardiotoxic agents"|cardiotoxicity|"cardiac toxicity"|"cardiovascular toxicity" "aortic|arterial|artery|vascular stiffness|elasticity|stretching|compliance"|pulse wave velocity

***Clinical Trials databases (for ongoing clinical studies)***

**WHO International Clinical Trials Registry Platform**
<http://apps.who.int/trialsearch/default.aspx> (Advanced Search)

cancer OR tumor OR neoplasm OR carcinoma OR carcinogen OR malignant OR leukemia OR lymphoma OR sarcoma OR myeloma (in condition)
AND
anthracycline OR cardiotoxic agents OR cardiotoxicity OR cardiac toxicity OR cardiovascular toxicity (in intervention)

**Clinical Trials.gov**<https://clinicaltrials.gov/>

9 Studies found for: Cancer OR neoplasm OR carcinoma OR malignant OR tumor OR leukemia OR lymphoma OR sarcoma OR myeloma (in Condition or disease)| anthracycline OR cardiotoxic agents OR cardiotoxicity (in Intervention/treatment) | ((arterial OR aortic OR vascular) AND (stiffness OR elasticity OR remodelling)) OR pulse wave velocity (in Outcome Measure)

**Tips:**

- We recommend **citation tracking (backward and forward)** of included studies (Google Scholar or Web of Science).
- **Check** all included studies for possible **retractions**.

Please fill in the PRISMA Flow diagram:
<http://prisma-statement.org/prismastatement/flowdiagram.aspx>
